# Refractory Diarrhea After Dor Fundoplication: The Long-Term Impact of Proton Pump Inhibitor Therapy

**DOI:** 10.7759/cureus.84301

**Published:** 2025-05-17

**Authors:** Kyle Mangum, Mark Bazemore, Jeannette Sandoval, Nathan Kragh, Basem Soliman

**Affiliations:** 1 School of Medicine, Texas Tech University Health Sciences Center, Amarillo, USA; 2 Department of Surgery, Texas Tech University Health Sciences Center, Amarillo, USA

**Keywords:** gerd, hiatal hernia, partial fundoplication, proton pump inhibitor, refractory diarrhea

## Abstract

Persistent diarrhea following hiatal hernia repair is a rare but noteworthy postoperative complication. In this case, we present a 62-year-old female with a longstanding history of gastroesophageal reflux disease (GERD) responsive to proton pump inhibitors (PPIs). After undergoing robotic-assisted hiatal hernia repair with partial fundoplication, the patient discontinued PPI therapy and subsequently developed chronic diarrhea. Extensive testing, including stool cultures, returned negative, and antidiarrheal medications provided minimal relief. Interestingly, reintroduction of PPI therapy resulted in a complete resolution of symptoms within days. This case suggests a possible correlation between cessation of long-term PPI use and gastrointestinal dysfunction, with chronic diarrhea manifesting as a withdrawal-like effect. Further investigation into the role of PPI discontinuation on gut microbiota and its clinical outcomes is necessary to understand this phenomenon better.

## Introduction

The widespread use of proton pump inhibitors (PPIs) in managing gastroesophageal reflux disease (GERD) is common in the United States, with concerns about overuse [1]. Long-term PPI use can lead to increased susceptibility to infections, secondary hypergastrinemia, impaired absorption of micronutrients, and idiosyncratic reactions [2]. Definitive treatment for GERD should be pursued where possible, especially in patients who do not achieve symptom control with lifestyle modifications and medical therapy alone [3]. Patients with hiatal hernias may benefit from surgical intervention and often can discontinue PPI therapy postoperatively [4]. However, the sequelae of long-term PPI use can contribute to postoperative complications when medication is discontinued [5]. In this report, we present a case of a patient with a 20-year history of GERD that was well controlled with daily PPI therapy who developed chronic diarrhea after Dor fundoplication and cessation of PPI therapy.

## Case presentation

**Figure 1:**
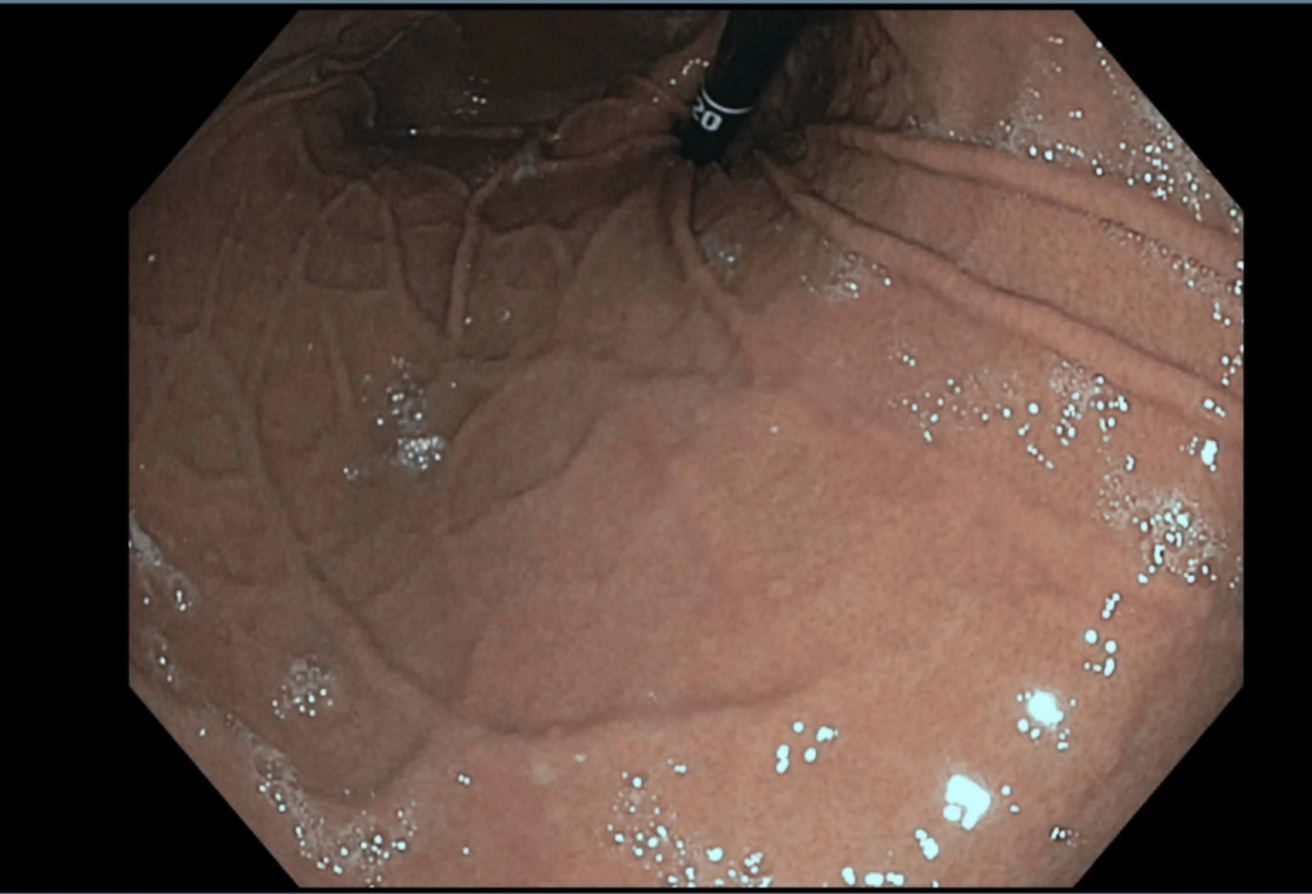
Esophagogastroduodenoscopy image of the paraesophageal hernia.

A 62-year-old female presented for the first time with a referral for a screening colonoscopy. She had no history of prior colonoscopies and denied any blood in her stool, changes in bowel habits, unexplained weight loss, or abdominal pain. The patient reported a 20-year history of GERD adequately managed with daily PPI therapy. Her past medical history included acid reflux, atrial fibrillation, benign essential hypertension, depression, overactive bladder, and a history of a non-ST elevation myocardial infarction (NSTEMI) two years prior. She had a surgical history of cholecystectomy and hysterectomy. The patient underwent a screening colonoscopy as well as an esophagogastroduodenoscopy (EGD) due to long-standing GERD. The EGD revealed a small paraesophageal hernia (PEH) but no significant gastritis, and the colonoscopy was unremarkable (Figure 1). Medical and surgical management were discussed, and she opted for medical management.

**Figure 2:**
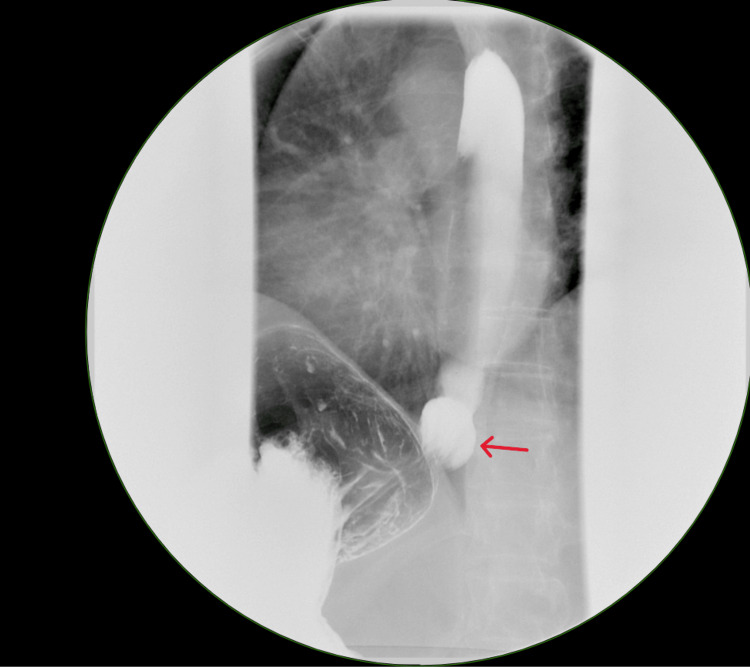
Hiatal hernia on esophagram.

**Figure 3:**
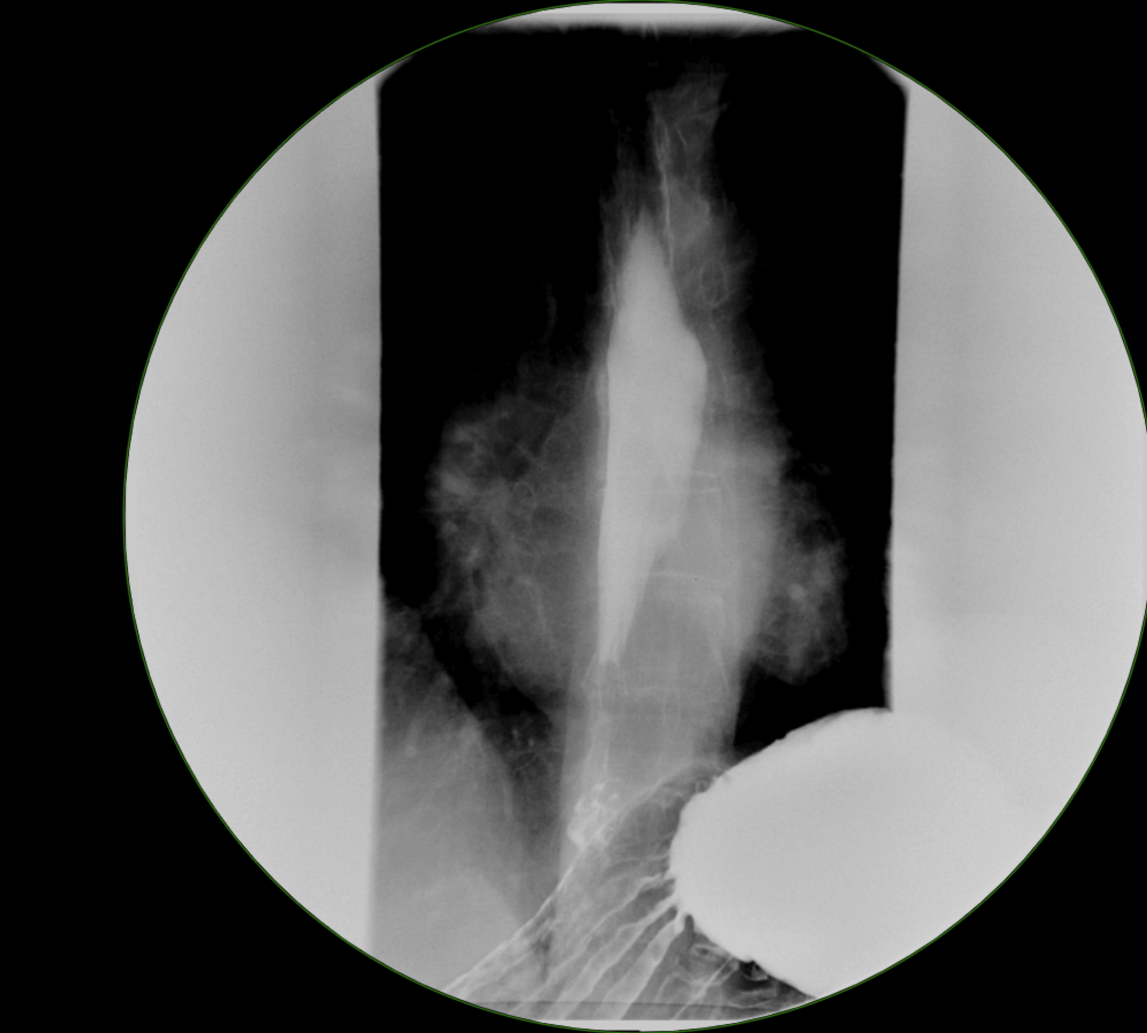
Gastric reflux on esophagram.

Four months later, the patient expressed interest in surgery to resolve her long-standing GERD symptoms. An esophagram was ordered, revealing a small hiatal hernia (Figure 2) and moderate reflux (Figure 3). After discussing surgical options, the patient opted for a robotic hiatal hernia repair with mesh and fundoplication. The operation proceeded as planned, with no complications. She was discharged on postoperative day one. Two weeks after her fundoplication, the patient presented for follow-up. She reported recovering well with minimal pain, no nausea or vomiting, tolerating a soft diet, and regular bowel function. The plan included continued dietary and lifting restrictions for a short period, and otherwise was to follow up as needed.

Approximately one month later, the patient presented to the clinic with complaints of chronic diarrhea and lower abdominal pain since the surgery. She denied fever, nausea, vomiting, or dysphagia. A stool panel was done and was negative. The patient had not taken her PPI since the surgery, which raised suspicion of a possible intestinal flora overgrowth or infectious etiology. Trials of cholestyramine and Imodium were utilized with short follow-ups in the clinic. She continued with ongoing, persistent complaints of chronic diarrhea. She reported variable frequency of diarrhea, sometimes severe, and admitted to a high intake of dairy products. Her dietary habits, particularly the consumption of ice cream, were considered potential triggers. She was educated and instructed on trials of dietary restrictions and was started on a probiotic. We discussed the possibility that stopping the PPI postoperatively could have led to gut microbiome dysbiosis due to the abrupt changes in gut pH. In the coming weeks, the patient returned for persistent daily diarrhea with nighttime episodes that would wake her. She was to the point that she avoided social situations due to urgency and incontinence. Patient was compliant with her probiotics and held a strict non-dairy diet with no relief of symptoms. She was restarted on omeprazole 40 mg daily for the next two weeks. Two weeks later, the patient presented with complete resolution of her diarrhea. She stated that a couple of days after restarting her omeprazole, her symptoms completely resolved, and her bowel movements were solid and regular. The patient was feeling well overall. Six weeks later, she returned to the clinic with continued resolution of her symptoms.

## Discussion

As the side effects of long-term PPI use become better understood, anti-reflux surgery becomes more attractive [6]. However, these surgeries are not without risk. Studies have examined complications from fundoplication, with diarrhea being a notable one [7]. Reported rates vary by study and postoperative timing. One study showed that 9% of patients experienced diarrhea for up to three months after surgery, while only 1% had it during the 3-to-12-month period [8]. Another study found that 15% of patients reported new-onset diarrhea postoperatively, with most experiencing symptoms for at least two years [9].

Three potential theories have been proposed to explain post-fundoplication diarrhea: dumping syndrome, vagal nerve injury, and exacerbation of pre-existing irritable bowel syndrome (IBS) [10]. While vagal injury [11] and dumping syndrome [12] related to fundoplication have been further explored, these explanations do not fit this case well. Our patient had no pre-existing IBS symptoms, and her diarrhea resolved after reintroducing PPI therapy, which seems unrelated to vagal injury or dumping syndrome. The resolution of symptoms with omeprazole, alongside negative stool analysis and a 14-week duration of diarrhea resistant to antidiarrheal therapy, supports the theory that PPI discontinuation contributed to her complications.

Complications following PPI cessation are not uncommon. In 2022, the American Gastroenterological Association released guidelines for PPI discontinuation, citing overuse and potential cessation-related complications [13]. Current literature focuses on rebound acid hypersecretion and its effects on patients stopping PPI therapy. PPI use has also been shown to alter gastric and fecal microbiota, though the clinical consequences remain unclear [14]. In this case, we hypothesize that chronic PPI use altered the patient's microbiome to the point that normal or hypersecretion of gastric acid caused gastrointestinal dysfunction, manifesting as resistant diarrhea. It is also possible that interactions between PPI cessation and the fundoplication surgery contributed to her complications.

## Conclusions

This case report highlights that diarrhea following hiatal hernia repair may be related to discontinuation of PPI therapy rather than solely a complication of the surgery itself. This may be especially true for patients whose diarrhea persists beyond the three-month postoperative period. Further research is needed on the effects of PPI discontinuation on the gut microbiome and its clinical ramifications. Physicians managing patients with post-fundoplication complications should consider restarting PPI therapy when significant postoperative side effects last longer than three months.
